# DNAzymes and Oligodeoxyribonucleotides Immobilized on Aminopropylsilanol and Polylysine‐Anatase Nanoparticles as Active Agents Against Influenza A/H5N1 Virus

**DOI:** 10.1111/cbdd.70419

**Published:** 2026-10-16

**Authors:** Marina Repkova, Asya Levina, Ekaterina Filippova, Elena Makarevich, Maria Protsenko, Larisa Shishkina, Artemiy Sergeev, Valentina Zarytova

**Affiliations:** ^1^ Institute of Chemical Biology and Fundamental Medicine, SB RAS Novosibirsk Russia; ^2^ FBRI State Research Center of Virology and Biotechnology “Vector” of Rospotrebnadzor Koltsovo Novosibirsk Region Russia

**Keywords:** anatase and aminopropylsilanol nanoparticles, DNAzymes, inhibition of influenza A/H5N1 virus, nanocomposites, oligodeoxyribonucleotides

## Abstract

The nanocomposites based on aminopropylsilanol (Si~NH_2_) and polylysine‐anatase (Ans/PL) nanoparticles bearing DNAzymes (Dz) and oligodeoxyribonucleotides (ON) can effectively inhibit the replication of the influenza A/H5N1 virus in cell culture. Nanocomposites based on both types of nanoparticles exhibit high activity. The Dz‐containing nanocomposites exhibited higher antiviral activity than those containing ON, reducing influenza A/H5N1 virus production by 3.5 log versus 2.4–3.0 log, respectively. Notably, the effect of Dz incorporated into the nanoparticles was an order of magnitude higher than that of unbound Dz transfected with lipofectamine. Nanocomposites carrying control DNA fragments that are not specific to the influenza A virus genome have practically no antiviral activity, indicating the specificity of the proposed nanocomposites. It was demonstrated that the antiviral efficacy of the nanocomposites is primarily determined by the activity of DNA delivered into cells, which can be varied by altering either the nanocomposite concentration or its capacity for DNA fragments. All of the proposed nanocomposites can be considered as potential antiviral drugs.

## Graphical abstract

Scheme of localization of DNAzymes (Dz) and oligonucleotides (ON) on segment 5 of the influenza A/H5N1 virus (A). The nanocomposites based on aminopropylsilanol (Si) and anatase (Ans) nanoparticles bearing Dz and ON effectively inhibit the virus replication in the infected MDCK cells by 2.5–3.5 lg (B).

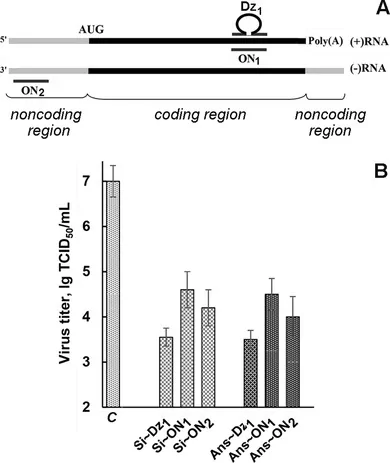

**Abbreviations**
Ans: TiO_2_ nanoparticles in the anatase form
Dz: DNAzyme
IAV: influenza A virus
ON: oligodeoxyribonucleotide
PL: polylysine
Si~NH_2_
: aminopropylsilanol nanoparticles

## 1 Introduction

Nucleic acid (NA) fragments (RNA and DNA fragments, their analogues, DNAzymes, RNAzymes, aptamers, small interfering RNAs (siRNAs), etc.) are often used in the development of methods for effective and selective action on the intracellular genome. NA fragments can recognize mRNA sequences of certain genes, block their function, and thus inhibit the expression of undesirable genes that cause various genetic, viral, and oncological diseases. These therapeutic agents should be as effective as possible with minimal side effects.

All nucleic acid fragments act by interacting with complementary regions of mRNA of the target genes, which leads to either mechanical blocking of mRNA or its degradation with the participation of special proteins. In both cases, the expression of the corresponding genes is inhibited (Crooke et al. 2021; Shen and Corey 2018). Catalytically active nucleic acids, DNAzymes, are of great interest. They can form a secondary structure adapted to the target RNA and cleave it catalytically, as a kind of gene‐specific molecular scissors (Franzen 2010).

Using nanoparticles as NA‐based drug delivery vehicles is a promising approach (Weng et al. 2020). Titanium dioxide nanoparticles have been identified as potential NA fragment carriers due to their ability to penetrate the plasma membrane, stability, and the possibility of surface modification to immobilize various compounds (Thurn et al. 2011). The cytotoxicity of titanium dioxide nanoparticles is manifested at high concentrations (more than 200 μg/mL), whereas at lower concentrations, they are nontoxic (Jeng and Swanson 2006; Ismagilov et al. 2012).

Silicon dioxide nanoparticles have shown promising potential as nonviral nanovectors for nucleic acid (NA) fragments. These nanoparticles are less toxic than the widely used transfection agent lipofectamine and can protect NA fragments from nucleases (Cheang et al. 2012; Lieberman et al. 2014).

Previously, we demonstrated the efficient inhibition of influenza A virus (IAV) replication by ON‐ and Dz‐containing nanocomposites based on TiO_2_ nanoparticles (e.g., Levina et al. 2016, 2024) and ON‐containing nanocomposites based on aminopropylsilanol nanoparticles (Levina et al. 2018).

In this paper, we report the first synthesis of Dz‐containing nanocomposites based on aminopropylsilanol nanoparticles, aiming to compare their antiviral activity against the influenza A/H5N1 virus with that of previously synthesized Dz‐ or ON‐containing formulations and to identify the most potent candidates.

## 2 Materials and Methods

### 2.1 Materials

We used chemicals from commercial suppliers: (3‐aminopropyl)triethoxysilane (APTES), penicillin–streptomycin (100 U/mL), poly‐L‐lysine hydrobromide (PL, MW 15,000–30,000) (Sigma‐Aldrich, USA); DMEM medium (Biolot, Russia); Lipofectamine 2000 (Invitrogen, USA). Chicken erythrocytes, MDCK cells, and influenza A virus strain A/chicken/Kurgan/05/2005 (H5N1) were from collection of FBRI SRC VB “Vector” Rospotrebnadzor, Russia. Dz and ON were synthesized by the solid‐phase phosphoramidite method on an automatic DNA ASM‐800 synthesizer (Biosset, Russia) using nucleoside phosphoramidites (Glen Research, USA). The ON and Dz concentration was measured by their optical absorption on a Shimadzu U‐1800 spectrophotometer (Shimadzu, Japan).

### 2.2 Preparation of Ans, Ans/PL, and Si~NH_2_ Nanoparticles and Dz‐ and ON‐Containing Nanocomposites

Aminopropylsilanol nanoparticles (Si~NH_2_) were synthesized by hydrolysis of (3‐aminopropyl)triethoxysilane (APTES) (Levina et al. 2018). In short, APTES was added dropwise to hot (70°C) water, followed by stirring the mixture at this temperature for 15 h and cooling then to the room temperature. The resulting product is a clear aqueous solution of low‐molecular polymer chains consisting of the [–Si(OH)(C_3_H_6_NH_2_)O−] monomer units. The Si~NH_2_•ON nanocomposites were prepared through the electrostatic interaction between the negatively charged internucleotide phosphate groups and positively charged amino groups in nanoparticles by mixing Si~NH_2_ and ON in saline solution or water for 1 h at room temperature. The binding of ON to Si~NH_2_ was confirmed by electrophoresis in agarose gel (Levina et al. 2018).

The Si~NH_2_•Dz nanocomposites were obtained in this work by analogous procedure by mixing 0.3 M Si~NH_2_ (2 μL, 0.08 mg) and 10^−4^ M Dz (3 μL) in 0.16 M NaCl or water (98 μL). The molar ratio of the amino groups in the nanoparticles to the phosphate groups in an oligonucleotide was NH_2_/*p* ~ 50 to provide the quantitative formation of the Si~NH_2_•Dz nanocomposites. After stirring the mixture for 1 min, it was left at room temperature for 30 min to facilitate complexation. The capacity of the nanocomposites for Dz was ~4 nmol/mg. The size and zeta potential values of aminopropylsilanol nanoparticles were measured by the dynamic light scattering (DLS) method on a Zetasizer Nano ZS Plus instrument (Malvern, UK). The zeta potential values of the samples were evaluated by phase analysis light scattering using DTS1070 cuvette.

Preparation of Ans nanoparticles is described in Levina et al. (2013). In short: a mixture of titanium tetraisopropoxide and isopropanol was added to the aqueous solution of HNO_3_ under intense stirring. The sol was stirred for 7 h at 70°C and cooled then to room temperature. After dialysis against deionized water, glycidyl isopropyl ether was added to the sample followed by the addition of LiOH to pH 9.5. Ans samples were subjected to dialysis against deionized water. Nanosols were stable for several months at 4°C.

Ans covered with polylysine (Ans/PL) were synthesized as described in (Repkova et al. 2024). In short: the aqueous PL solution was intensively mixed with Ans nanoparticles for 30 min. Ans/PL conjugates were prepared at a concentration of 1 mg/mL with the capacity of ~2 μmol of the amino groups per 1 mg of Ans.

The Ans/PL•ON and Ans/PL•Dz nanocomposites were obtained as described in (Levina et al. 2013, 2024) by mixing aqueous solutions of Ans/PL (0.1 mg for nanoparticles) and ON or Dz (2 nmol) for 30 min. The nanocomposites were prepared in near‐quantitative yields (90%–100%) with an ON and Dz loading capacity of 20 nmol/mg, unless otherwise specified.

The prepared nanoparticles and nanocomposites were characterized using physico‐chemical methods: IR and electron spectroscopy, inductively coupled plasma mass spectrometry (ICP‐MS), X‐ray diffraction (XRD), Scanning Electron Microscopy (SEM), Small‐Angle X‐ray Scattering (SAXS), Raman Spectroscopy, Atomic Force Microscopy (AFM), High‐Resolution Transmission Electron Microscopy (HR‐TEM) (Levina et al. 2013, 2018).

### 2.3 Evaluation of 50% Toxic Concentration (CT_50_ ) for Si~NH_2_ •Dz

MDCK cells in logarithmic phase were seeded at 100,000 cells/mL in DMEM nutrient medium in 96‐well plates (100 mL/well) and incubated at 37°C and 5% CO_2_. Two days after the formation of a continuous mono layer of MDCK cells at 37°C and 5% CO_2_, the cells were washed with nutrient medium without serum. The Si ~ NH_2_•Dz sample was diluted with DMEM medium to the needed concentration (2–30 μM for Dz) and incubated with MDCK cells at 37°C and 5% CO_2_ for 2 days. Destructive changes in the cells were evaluated by an inverted microscope. MDCK cells without studied samples were used as a control and taken for 100%. The cells were stained with trypan blue (Altman et al. 1993), and the number of viable cells was counted in a Goryaev chamber.

### 2.4 Antiviral Activity of the Proposed Nanocomposites

The A/chicken/Kurgan/05/2005 virus (H5N1) was grown in the allantoic cavity of 10‐day‐old embryonated chicken eggs at 37°C. Allantoic fluid was harvested for 48 h after virus inoculation, aliquoted, and stored at −80°C. The MDCK cells were seeded at 100,000 cells/mL in the DMEM nutrient medium that contained 10% fetal calf serum (Gibco, USA) in 96‐well plates (100 μL/well) and incubated at 37°C, 5% CO_2_, and 100% humidity. After reaching ~80% confluence, the medium was removed, and the samples of the studied nanocomposites at 0.1 μM concentration for Dz or ON (5 μg/mL for Ans and 25 μg/mL for Si~NH_2_) in 100 μL of the DMEM medium were added to the wells. The cells in the same medium without a sample were used as the control sample. The cells were incubated at 37°C, 5% CO_2_, and 100% humidity for 4 h, followed by washing the cells with the same medium. The cells were then infected with the A/H5N1 virus in the DMEM medium (100 μL in each well) at a MOI of 0.01 TCID_50_/cell. After the virus adsorption for 1 h at room temperature, the virus‐containing medium was removed, cells were rinsed with the DMEM medium, and the same medium was added to each well (100 μL). After incubation for 48 h, serial 10‐fold dilutions (from 10^−1^ to 10^−8^) of the cultural virus‐containing liquid from each well were applied to MDCK cells for 48 h to evaluate the virus titer. The cytopathic action of the virus was revealed under a microscope and in the hemagglutination reaction with a 1% suspension of chicken erythrocytes. The virus titer was expressed in terms of logTCID_50_/mL.

The dependence of the virus titer on the ON concentration in the nanocomposites (μM) was studied in two variants. (1) The capacity of the nanocomposite for ON varied at a constant concentration of the nanocomposites. In the case of Ans/PL•ON, the ON concentration varied from 0 to 80 nmol/mg at a constant nanocomposite concentration of 5 μg/mL. In the case of Si~NH_2_•ON, the ON concentration varied from 0 to 16 nmol/mg at a constant nanocomposite concentration of 25 μg/mL. (2) The concentration of nanocomposites varied at a constant ON capacity. In the case of Ans/PL•ON, the nanocomposite concentration varied from 0 to 20 μg/mL with a constant ON capacity of 20 nmol/mg. In the case of Si~NH_2_•ON, the nanocomposite concentration varied from 0 to 100 μg/mL at a constant ON capacity of 4 nmol/mg.

### 2.5 Statistical Analysis

Statistical analysis was carried out using the Statistica 7.0 program. The titer of the virus in the control (without experimental samples) and experiment (in the presence of experimental samples) was calculated using the Spearman–Karber method and expressed in logarithms of 50% of tissue cytopathic doses per mL (logTCID_50_/mL). The differences between the results with experimental and control samples were considered statistically significant at *p* ≤ 0.05.

## 3 Result and Discussion

We chose segment 5 of the influenza A virus as a target for exposure to nanocomposites because it encodes a nucleoprotein (NP) that plays a key role in incorporating the viral genome into the cells of an infected organism. This nucleoprotein also promotes the replication and assembly of viral particles (Portela and Digard 2002; Hu et al. 2017). The ON and Dz sequences used in this study are presented in Table 1.

**TABLE 1:** DNA fragments for creation of nanocomposites.

Code	Nucleotide sequence (5′➔3′)	Aimed at IAV segment 5
Dz_1_	GAAATAAGAggctagctacaacgaCCTTCATTA^a^	(+)RNA (region 1490–1509)^b^
ON_1_	GAAATAAGAtCCTTCATTA^a^	(+)RNA (region 1490–1509)^b^
ON_2_	GCAAAAGCAGGGTAGATAATC	(−)RNA (region 2–22)^b^
сDz^c^	TCAACTCCAggctagctacaacgaATGCCATGT^a^	Control Dz
cON^c^	AATCAAGTCCATGTGCCATGT	Control ON

^a^ Central catalytic fragment of Dz (lowercase letters) is instead of the nucleotide unit (t) in corresponding ON_1_.

^b^ The number of nucleotides is given for (+)RNA.

^c^ сDz is the control DNAzyme with a standard catalytic domain and the flanks nonspecific for IAV, сON is the control oligodeoxyribonucleotide complementary to the bovine diarrhea virus (strain Oregon C24V, GenBank).

Dz of the “10–23” type composed of a central catalytic fragment (lowercase letters) and flanks complementary to the coding region of (+)RNA of IAV segment 5 exhibited the highest activity among a large set of DNAzymes that target various regions of the influenza virus genome (Evdokimov et al. 2013). ON_1_ is targeted to the same region as Dz_1_. ON_2_ targeted to the noncoding region of (−)RNA of IAV segment 5 was chosen because it was shown to exhibit the maximal antiviral activity compared to the other studied ONs (Levina et al. 2016). DNA fragments cON and cDz, which are not complementary to the IAV genome, were used as controls (Table 1).

We used nanoparticles of aminopropylsilanol (Si~NH_2_) (Levina et al. 2018) and crystalline anatase titanium dioxide nanoparticles coated with polylysine (Ans/PL) (Levina et al. 2013) to create nanocomposites carrying DNA fragments.

The Si~NH_2_•Dz nanocomposites were prepared by immobilizing Dz to the nanoparticles through electrostatic binding of internucleotide phosphates of Dz with the amino groups in nanoparticles. The size of the resulting product increased up to ~3.5 nm compared to initial nanoparticles (~1 nm). The addition of negatively charged Dz molecules to Si ~ NH_2_ led to a change in the zeta potential from ~(+9) mV on the initial nanoparticles up to ~(−40) mV. The value of the zeta potential of Si~NH_2_ is positive due to positively charged amino groups in neutral medium. When the nanoparticles were bound to Dz, the zeta potential value, as expected, became negative due to the negative charge of Dz. The changes in the size and zeta potential indicate the formation of the nanocomposite Si~NH_2_•Dz. These data correlate with those of the previous work for Si~NH_2_•ON (Levina et al. 2018).

The Si~NH_2_•ON, Ans/PL•Dz, and Ans/PL•ON nanocomposites were prepared by immobilizing the chosen Dz and ON to the corresponding nanoparticles. Characterization of the prepared nanoparticles and nanocomposites was performed using physico‐chemical methods (Levina et al. 2013, 2018, 2024).

The Si~NH_2_ nanoparticles and Si~NH_2_•ON nanocomposites were characterized in (Levina et al. 2018). According to the TEM images, there is a difference in morphology between Si~NH_2_ nanoparticles and their complexes with ON. Discrete nanometer‐sized nanoparticles are quite homogeneous, whereas Si~NH_2_•ON forms chain‐like structures. The Si/P weight ratio for the analyzed Si~NH_2_•ON sample evaluated by inductively coupled plasma mass spectrometry (ICP‐MS) showed a reasonable correlation between experimental and calculated values. The very small size of the Si~NH_2_ particles and corresponding nanocomposites allows for the formation of true solutions. Si~NH_2_ and Si~NH_2_•ON can be stored for a long time (at least for a month at 25°C and 3 months at 4°C) without changes in size, which indicates no aggregation of these preparations.

The Ans nanoparticles and Ans/PL•ON nanocomposites were characterized in (Levina, Ismagilov, et al. 2012; Levina et al. 2013). The phase composition of Ans nanoparticles was confirmed by the XRD and IR methods. Average particle size of anatase nanoparticles defined by SAXS and AFM was 5–6 nm. HR‐TEM showed that the size of the Ans/PL•ON nanocomposites was broader by ~1 nm than that of the original nanoparticles, which indicated the formation of complexes between DNA fragments and nanoparticles. The phosphate to titanium ratio (Ti/P) in the nanocomposites, evaluated by the SEM study including the elemental analysis of nanocomposite surface by the EDAX spectrometer coupled with a scanning microscope, showed a good correlation between calculated and experimental data (Levina et al. 2018).

Using radioactively and fluorescently labeled oligonucleotides, we confirmed their attachment to nanoparticles and showed that the oligonucleotides in the nanocomposites retained their ability to form complementary complexes.

Immobilization of the oligonucleotides on the nanoparticles in water resulted in the formation of non‐agglomerated small‐sized monodisperse particles (~29 nm for Ans/PL and ~33 nm for Ans/PL•ON). Ans/PL and Ans/PL•ON nanocomposites did not change their hydrodynamic diameter and particle size distribution during at least 3 months. In normal saline or cell culture medium, the Ans/PL‐based nanocomposites are prone to agglomeration. Therefore, they were prepared and stored in water before use (Repkova et al. 2024). The activity of the sample prepared in water did not change during at least 10 days of storage.

The created nanocomposites were shown to penetrate eukaryotic cells (Levina et al. 2018; Repkova et al. 2017).

We studied the dose‐dependent antiviral activity of ON‐containing nanocomposites based on Ans/PL or Si~NH_2_ nanoparticles by varying either the nanocomposite concentration (mg/mL) at a constant ON capacity (nmol/mg) or the ON capacity at a constant nanocomposite concentration (Figure 1). Both variants resulted in a change in the ON concentration (μM).

**FIGURE 1:**
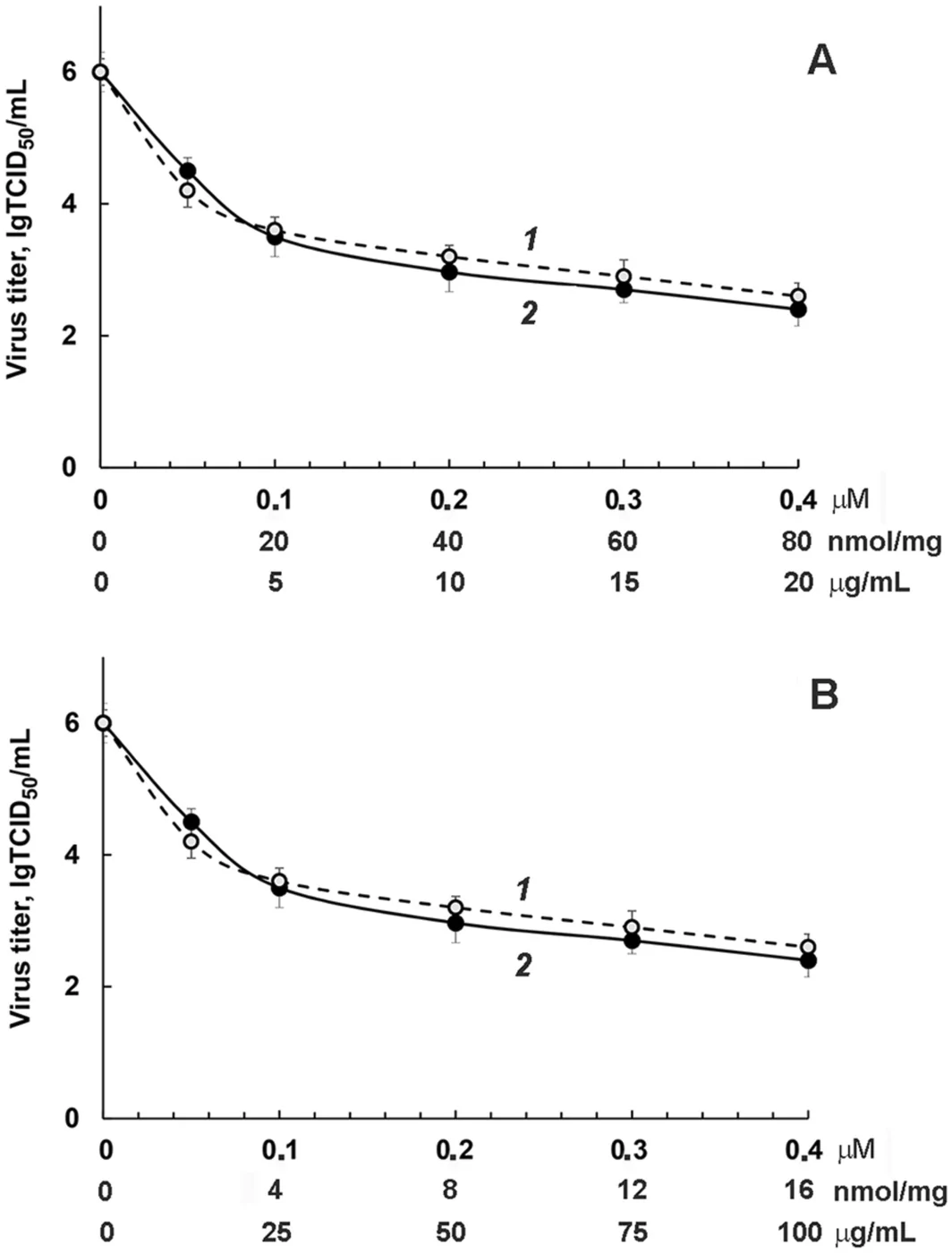
Dependence of influenza virus replication (titers) on capacity and concentration of Ans/PL•ON (A) or Si~NH_2_•ON (B) nanocomposites. The multiplicity of infection is 0.01 TCID_50_/cell. The x‐axis represents either the nanocomposite concentration (μg/mL), or the ON capacity (nmol/mg), or the ON concentration in the nanocomposites (μM). In Figure 1A, the change of the Ans/PL•ON concentration from 0 to 20 μg/mL at the constant capacity of 20 nmol/mg for ON leads to the change of the ON concentration in the nanocomposite from 0 to 0.4 μM. The same variation of the ON concentration is caused by the change in the ON capacity in Ans/PL•ON from 0 to 80 nmol/mg at the constant nanocomposite concentration of 5 μg/mL. In Figure 1B, the change of the Si~NH_2_•ON concentration from 0 to 100 μg/mL at the constant capacity of 4 nmol/mg for ON leads to the change of the ON concentration in the nanocomposite from 0 to 0.4 μM. The same variation of the ON concentration is caused by the change in the ON capacity in Si~NH_2_•ON from 0 to 16 nmol/mg at the constant nanocomposite concentration of 25 μg/mL. Curves 1 in both figures reflect the dependence of the virus titer on the nanocomposite capacity for ON at a constant nanocomposite concentration. Curves 2 reflect the dependence of the virus titer on the concentration of nanocomposites at a constant ON capacity.

The coincidence of curves 1 and 2 in Figure 1A,B demonstrates that the antiviral efficacy of the nanocomposites is primarily determined by the active DNA delivered into cells, which can be varied by altering either the nanocomposite concentration (mg/mL) or its capacity for DNA fragments (nmol/mg).

The 0.1 μM ON concentration in the nanocomposite was selected as optimal for further studies, as the dose–response curve almost reached a plateau, and a further increase in the ON concentration yielded minimal additional antiviral effect. The same conclusion was made in (Levina, Repkova, et al. 2012; Levina et al. 2024) while performing the dose dependence studies of the in vitro antiviral activity of the Ans/PL•ON_2_ and Ans/PL•Dz_1_ nanocomposites against A/H5N1 virus.

We evaluated the 50% cytotoxic concentration (TC_50_) of the Si~NH_2_•Dz_1_ nanocomposite against MDCK cells, which exceeded 20 μM for Dz_1_. This finding is consistent with the TC_50_ values previously reported for Si~NH_2_•ON, Ans/PL•ON, and Ans/PL•Dz (Levina et al. 2014, 2018, 2024). It has also been shown that the toxicity of the nanocomposites depends solely on the nanoparticles. To evaluate antiviral activity against the A/H5N1 virus, the nanocomposites were applied at a non‐toxic concentration (0.1 μM for ON or Dz in the nanocomposites), well below their respective TC_50_ thresholds.

The localization of complementary ON and Dz on segment 5 of the influenza A virus is schematically shown in Figure 2A. The results of the antiviral activity of the created nanocomposites are shown in Figure 2B. The nanocomposites bearing ON or Dz targeted to the viral genome exhibited high activity against the A/H5N1 virus (columns 7 vs. 10, 8 vs. 11, and 9 vs. 12). Control samples (Ans/PL and Si~NH_2_ particles and free ON or Dz) exhibited insignificant antiviral activity (data not shown). Nanocomposites bearing the control samples cDz and cON (columns 1–4), which are not specific to the influenza A virus genome, were also almost inactive. In contrast, all nanocomposites containing complementary DNA fragments (columns 7–12) selectively inhibited virus replication by 2.8–3.5 log. Complementary ON и Dz in the nanocomposites showed greater activity than unbound DNA fragments in the presence of the transfection agent lipofectamine (columns 5 and 6). Thus, we can conclude that Ans/PL and Si~NH_2_ nanoparticles turned out to be more efficient vehicles for delivering DNA fragments into cells. The Dz‐containing nanocomposites were more active than the ON‐containing nanocomposite by ~1 log when Dz_1_ and ON_1_ targeted the same region of the viral mRNA (columns 7 vs. 8 and 10 vs. 11). A slightly smaller difference in activity was observed between the activity of the nanocomposites containing Dz_1_ and ON_2_ that target different regions of the viral genome (columns 7 vs. 9 and 10 vs. 12).

**FIGURE 2:**
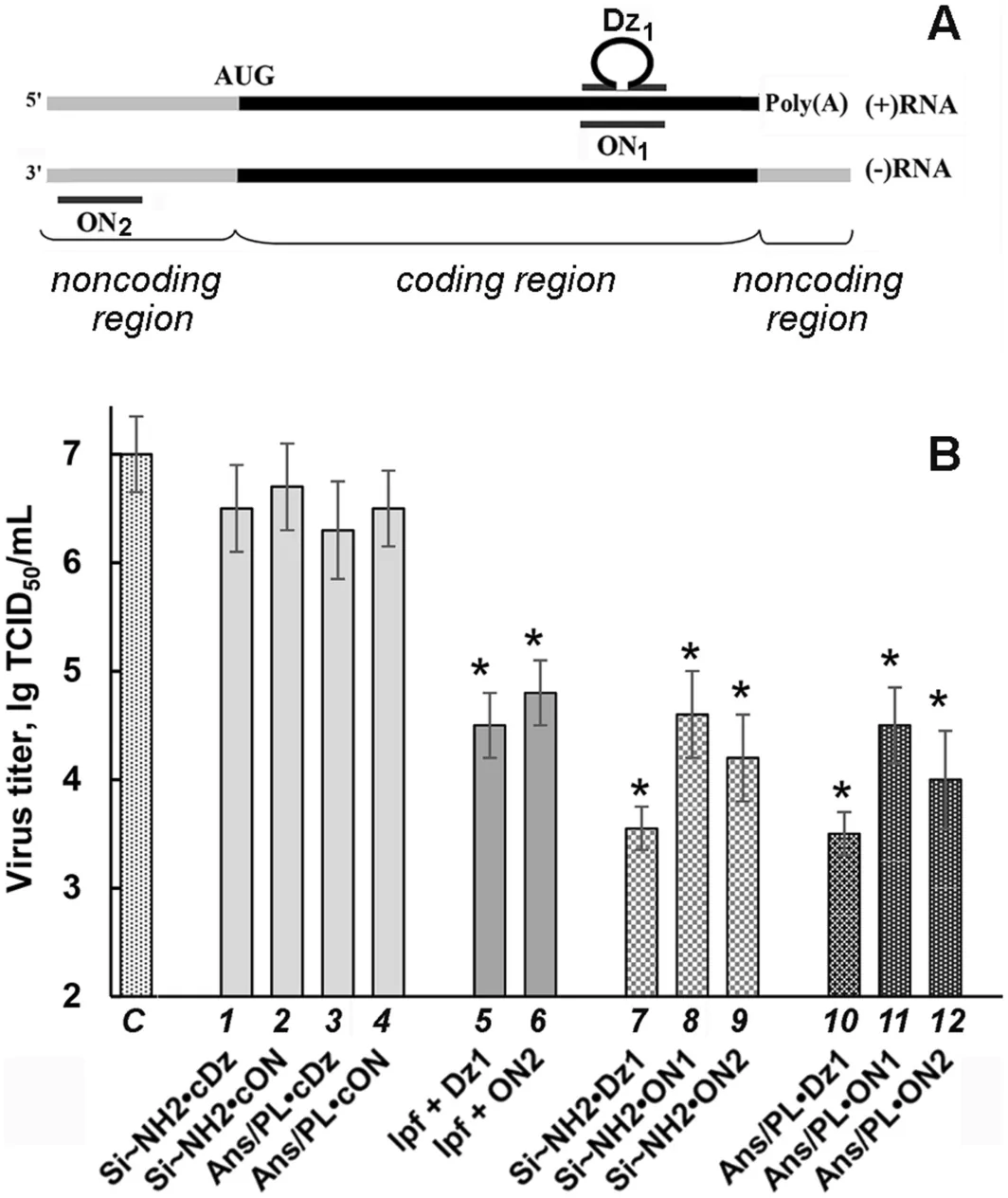
(A) Scheme of localization of the used DNA fragments on segment 5 of the influenza A virus. AUG is the initiating codon for the translation of the nucleoprotein gene. (B) Inhibition of influenza A/H5N1 virus production in infected MDCK cells using Dz or ON in nanocomposites based on Si~NH_2_ or Ans/PL nanoparticles. The concentrations of Dz or ON, Ans, and Si~NH_2_ in the samples were 0.1 μM, 25 μg/mL, and 5 μg/mL, respectively. C, virus control in the absence of samples. Nanocomposites contained either complementary (Dz_1_, ON_1_, or ON_2_) or non‐complementary (cDz or cON) DNA fragments for IAV genome. Dz_1_ and ON_2_ in the presence of lipofectamine (lpf) were used for the comparison. The multiplicity of infection was 0.01 TCID_50_/cell. The average values, standard deviations and differences in the titer values of the virus were calculated using the Spearman–Kerber method. *р ≤ 0.05 compared to the control.

Many researchers have described the use of ON and Dz against various subtypes of the influenza A virus. It should be noted that they used transfection agents to deliver the active molecules into the cells. Using antisense ON (4 μM), a decrease in virus titer by 1.0–1.2 log was demonstrated (Zhang et al. 2011). Morpholine oligonucleotides conjugated with a delivery peptide were effective against the influenza A virus, with an inhibitory effect of 2.0 log at a concentration of 30 μM (Ge et al. 2006). DNAzymes that target the M1, M2, and PB2 genes of the influenza A/PR/8/34 virus, when delivered to cells using lipofectamine, inhibited viral replication by 70% and 99% at a concentration of 0.5 μM and 10 μM (Kumar et al. 2015). We achieved a significant antiviral effect, inhibiting virus production by 3.5 log (99.97%) using the proposed Si~NH_2_•Dz and Ans/PL•Dz nanocomposites at a significantly lower Dz concentration (0.1 μM) with no additional effects on cell transfection.

## 4 Conclusion

For the first time, Dz‐containing nanocomposites based on aminopropylsilanol (Si~NH_2_) nanoparticles have been synthesized, and their antiviral activity against the influenza A/H5N1 virus has been evaluated and compared with that of other Dz‐ or ON‐containing nanocomposites.

Nanocomposites bearing DNA fragments targeted to the influenza A/H5N1 virus genome significantly suppressed viral replication in vitro by 2.8–3.5 log (a 99.83%–99.97% reduction), demonstrating their exceptionally high potency. Conversely, nanocomposites carrying noncomplementary DNA fragments exhibited negligible activity. This clear contrast confirms the sequence‐specific mechanism of the proposed nanocomposites.

It was demonstrated that the antiviral efficacy of the nanocomposites is primarily determined by the activity of DNA delivered into cells, which can be varied by altering either the nanocomposite concentration or its capacity for DNA fragments.

Our results clearly demonstrate that the antiviral effect of the proposed nanocomposites is directly attributable to their sequence‐specific inhibitory action rather than non‐specific toxicity, as the concentration used was well below their TC_50_ values.

The selected Dz molecule delivered to cells in the presence of lipofectamine exhibited lower activity compared to the same Dz incorporated into nanoparticles. Consequently, both anatase and aminopropylsilanol nanoparticles demonstrated superior efficiency as carriers for delivering DNA fragments into cells.

The Dz‐containing nanocomposites reduced viral replication by 3.5 log, which is 0.5–1.0 log more effective than the ON‐containing nanocomposites, particularly when both Dz and ON targeted the same region of the viral genome.

Consequently, all evaluated nanocomposite formulations represent promising antiviral candidates, allowing for a choice based on a balance between synthesis cost, simplicity, and specific therapeutic activity.

## Author Contributions

**Marina Repkova:** investigation, formal analysis, writing – original draft. **Asya Levina:** formal analysis, writing – original draft, writing – review and editing. **Ekaterina Filippova:** investigation, formal analysis. **Elena Makarevich:** investigation, formal analysis. **Maria Protsenko:** investigation, formal analysis. **Larisa Shishkina:** methodology, data curation. **Artemiy Sergeev:** methodology, data curation. **Valentina Zarytova:** conceptualization, data curation, supervision.

## Funding

This work was supported by the Russian state‐funded project for ICBFM SB RAS (grant number 125012300656‐5) and financing of the State Assignment no. 24/26‐3.3.1 of the State Research Сenter of Virology and Biotechnology “Vector”, Russia.

## Conflicts of Interest

The authors declare no conflicts of interest.

## Data Availability

The authors have nothing to report.

## References

[cbdd70419-bib-0001] Altman, S. A. , L. Randers , and G. Rao . 1993. “Comparison of Trypan Blue Dye Exclusion and Fluorometric Assays for Mammalian Cell Viability Determinations.” Biotechnology Progress 9: 671–674. 10.1021/bp00024a017. 7764357

[cbdd70419-bib-0002] Cheang, T. Y. , B. Tang , A. W. Xu , et al. 2012. “Promising Plasmid DNA Vector Based on APTES‐Modified Silica Nanoparticles.” International Journal of Nanomedicine 7: 1061–1067. 10.2147/IJN.S28267. PMC3292418 22403488

[cbdd70419-bib-0003] Crooke, S. T. , X.‐H. Liang , B. F. Baker , and R. M. Crooke . 2021. “Antisense Technology: A Review.” Journal of Biological Chemistry 296: 100416. 10.1016/j.jbc.2021.100416. PMC8005817 33600796

[cbdd70419-bib-0004] Evdokimov, A. A. , N. A. Mazurkova , E. G. Malygin , et al. 2013. “Design of Deoxyribozymes for Inhibition of Influenza A Virus.” Molekuliarnaia Biologiia (Mosk) 47, no. 1: 83–93. 10.7868/s0026898412060043. 23705497

[cbdd70419-bib-0005] Franzen, S. 2010. “Expanding the Catalytic Repertoire of Ribozymes and Deoxyribozymes Beyond RNA Substrates.” Current Opinion in Molecular Therapeutics 12: 223–232. 20373266

[cbdd70419-bib-0006] Ge, Q. , M. Pastey , D. Kobasa , et al. 2006. “Inhibition of Multiple Subtypes of Influenza A Virus in Cell Cultures With Morpholino Oligomers.” Antimicrobial Agents and Chemotherapy 50: 3724–3733. 10.1128/aac.00644-06. PMC1635187 16966399

[cbdd70419-bib-0007] Hu, Y. , H. Sneyd , R. Dekant , and J. Wang . 2017. “Influenza A Virus Nucleoprotein: A Highly Conserved Multifunctional Viral Protein as a Hot Antiviral Drug Target.” Current Topics in Medicinal Chemistry 17: 2271–2285. 10.2174/1568026617666170224122508. PMC5967877 28240183

[cbdd70419-bib-0008] Ismagilov, Z. R. , N. V. Shikina , E. V. Bessudnova , et al. 2012. “The Effect of Chemical Treatment Conditions of Titanium Dioxide Sols on Their Dispersion and Cytotoxic Properties.” Chemical Engineering Transactions 27: 241–246. 10.3303/CET1227041.

[cbdd70419-bib-0009] Jeng, H. A. , and J. Swanson . 2006. “Toxicity of Metal Oxide Nanoparticles in Mammalian Cells.” Journal of Environmental Science and Health, Part A 41: 2699–2711. 10.1080/10934520600966177. 17114101

[cbdd70419-bib-0010] Kumar, B. , R. Rajput , D. R. Pati , and M. Khanna . 2015. “Potent Intracellular Knock‐Down of Influenza A Virus M2 Gene Transcript by DNAzymes Considerably Reduces Viral Replication in Host Cells.” Molecular Biotechnology 57: 836–845. 10.1007/s12033-015-9876-z. 26021603

[cbdd70419-bib-0011] Levina, A. , Z. Ismagilov , M. Repkova , et al. 2012. “Nanocomposites Consisting of Titanium Dioxide Nanoparticles and Oligonucleotides.” Journal of Nanoscience and Nanotechnology 12, no. 3: 1812–1820. 10.1166/jnn.2012.5190. 22754985

[cbdd70419-bib-0012] Levina, A. S. , Z. R. Ismagilov , M. N. Repkova , et al. 2013. “TiO_2_~DNA Nanocomposites Capable of Penetrating Into Cells.” Bioorganicheskaia Khimiia 39: 87–98. 10.1134/S1068162013010068. 23844510

[cbdd70419-bib-0013] Levina, A. S. , M. N. Repkova , E. V. Bessudnova , E. I. Filippova , N. A. Mazurkova , and V. F. Zarytova . 2016. “High Antiviral Effect of TiO_2_•PL–DNA Nanocomposites Targeted to Conservative Regions of (−)RNA and (+)RNA of Influenza A Virus in Cell Culture.” Beilstein Journal of Nanotechnology 7: 1166–1173. 10.3762/bjnano.7.108. PMC5082348 27826491

[cbdd70419-bib-0014] Levina, A. S. , M. N. Repkova , Z. R. Ismagilov , et al. 2012. “High‐Performance Method for Specific Effect on Nucleic Acids in Cells Using TiO_2_~DNA Nanocomposites.” Scientific Reports 2: 756. 10.1038/srep00756. PMC3477653 23091696

[cbdd70419-bib-0015] Levina, A. S. , M. N. Repkova , Z. R. Ismagilov , N. V. Shikina , N. A. Mazurkova , and V. F. Zarytova . 2014. “Efficient Inhibition of Human Influenza a Virus by Oligonucleotides Electrostatically Fixed on Polylysine‐Containing TiO_2_ Nanoparticles.” Russian Journal of Bioorganic Chemistry 40, no. 2: 179–184. 10.1134/S1068162014020095. 25895339

[cbdd70419-bib-0016] Levina, A. S. , M. N. Repkova , N. A. Netesova , et al. 2024. “Substantial Antiviral Potential of Deoxyribozymes Fixed on Anatase Nanoparticles Against Influenza A Viruses In Vitro and In Vivo.” Journal of Pharmaceutical Sciences 113, no. 5: 1202–1208. 10.1016/j.xphs.2023.10.028. 37879408

[cbdd70419-bib-0017] Levina, A. S. , M. N. Repkova , N. V. Shikina , et al. 2018. “Non‐Agglomerated Silicon–Organic Nanoparticles and Their Nanocomplexes With Oligonucleotides: Synthesis and Properties.” Beilstein Journal of Nanotechnology 9: 2516–2525. 10.3762/bjnano.9.234. PMC6176811 30345214

[cbdd70419-bib-0018] Lieberman, A. , N. Mendez , W. C. Trogler , et al. 2014. “Synthesis and Surface Functionalization of Silica Nanoparticles for Nanomedicine.” Surface Science Reports 69: 132–158. 10.1016/j.surfrep.2014.07.001. PMC4212223 25364083

[cbdd70419-bib-0019] Portela, A. , and P. Digard . 2002. “The Influenza Virus Nucleoprotein: A Multifunctional RNA‐Binding Protein Pivotal to Virus Replication.” Journal of General Virology 83: 723–734. 10.1099/0022-1317-83-4-723. 11907320

[cbdd70419-bib-0020] Repkova, M. , A. Levina , B. Chelobanov , et al. 2017. “Efficient Inhibition of Influenza A Viral Replication in Cells by Deoxyribozymes Delivered by Nanocomposites.” International Journal of Antimicrobial Agents 49: 703–708. 10.1016/j.ijantimicag.2017.01.026. 28412273

[cbdd70419-bib-0021] Repkova, M. N. , O. Y. Mazurkov , E. I. Filippova , et al. 2024. “Non‐Agglomerated Oligonucleotide‐Containing Nanocomposites Based on Titanium Dioxide Nanoparticles.” Russian Journal of Bioorganic Chemistry 50, no. 6: 2634–2643. 10.1134/S1068162024060384.

[cbdd70419-bib-0022] Shen, X. , and D. R. Corey . 2018. “Chemistry, Mechanism and Clinical Status of Antisense Oligonucleotides and Duplex RNAs.” Nucleic Acids Research 46: 1584–1600. 10.1093/nar/gkx1239. PMC5829639 29240946

[cbdd70419-bib-0023] Thurn, K. T. , H. Arora , T. Paunesku , et al. 2011. “Endocytosis of Titanium Dioxide Nanoparticles in Prostate Cancer PC‐3M Cells.” Nanomedicine 7: 123–130. 10.1016/j.nano.2010.09.004. PMC3062699 20887814

[cbdd70419-bib-0024] Weng, Y. , Q. Huang , C. Li , et al. 2020. “Improved Nucleic Acid Therapy With Advanced Nanoscale Biotechnology.” Molecular Therapy ‐ Nucleic Acids 19: 581–601. 10.1016/j.omtn.2019.12.004. PMC6957827 31927331

[cbdd70419-bib-0025] Zhang, T. , T. Wang , P. Zhao , et al. 2011. “Antisense Oligonucleotides Targeting the RNA Binding Region of the NP Gene Inhibit Replication of Highly Pathogenic Avian Influenza Virus H5N1.” International Immunopharmacology 11: 2057–2061. 10.1016/j.intimp.2011.08.019. 21933722

